# Efficient Posterior Probability Mapping Using Savage-Dickey Ratios

**DOI:** 10.1371/journal.pone.0059655

**Published:** 2013-03-22

**Authors:** William D. Penny, Gerard R. Ridgway

**Affiliations:** Wellcome Trust Centre for Neuroimaging, Institute of Neurology, University College, London, United Kingdom; Banner Alzheimer’s Institute, United States of America

## Abstract

Statistical Parametric Mapping (SPM) is the dominant paradigm for mass-univariate analysis of neuroimaging data. More recently, a Bayesian approach termed Posterior Probability Mapping (PPM) has been proposed as an alternative. PPM offers two advantages: (i) inferences can be made about effect size thus lending a precise physiological meaning to activated regions, (ii) regions can be declared inactive. This latter facility is most parsimoniously provided by PPMs based on Bayesian model comparisons. To date these comparisons have been implemented by an Independent Model Optimization (IMO) procedure which separately fits null and alternative models. This paper proposes a more computationally efficient procedure based on Savage-Dickey approximations to the Bayes factor, and Taylor-series approximations to the voxel-wise posterior covariance matrices. Simulations show the accuracy of this Savage-Dickey-Taylor (SDT) method to be comparable to that of IMO. Results on fMRI data show excellent agreement between SDT and IMO for second-level models, and reasonable agreement for first-level models. This Savage-Dickey test is a Bayesian analogue of the classical SPM-F and allows users to implement model comparison in a truly interactive manner.

## Introduction

Bayesian inference has been applied to the analysis of fMRI data in multiple domains, ranging from connectivity analysis [1]–[4], group analysis [5], [6], haemodynamic modelling [7], spatial modelling [8], and state-space approaches [9], [10]. Generically, the advantage of these Bayesian approaches is that they allow for seamless incorporation of prior knowledge and employ established procedures for parameter regularization and model selection. Bayesian methods have also been widely used in the MEG/EEG domain for tackling the problems of source reconstruction [11], [12] and biologically informed connectivity analysis [13], [14]. The development and application of Bayesian methods to neuroimaging is described in recent reviews [15], [16]. The focus of this paper is a Bayesian method for the mass-univariate analysis of neuroimaging data, known as Posterior Probability Mapping (PPMs). Previously, PPMs have been proposed as a Bayesian alternative to Statistical Parametric Maps (SPMs) [17], [18]. PPMs can be applied to several common neuroimaging modalities (fMRI, PET, MEG, EEG) and provide estimates of effect size that are informed by empirical priors.

PPMs address a key limitation of classical frequentist inference: while a small p-value allows rejection of the null hypothesis, a large p-value does not permit its acceptance. Informally, absence of evidence is not evidence of absence. Bayesian model comparison, on the other hand, can find either the null or alternative hypothesis more probable [19], [20]. This enables imaging neuroscientists to infer that regions have not activated and so allows detection of double dissociations among brain regions and cognitive processes. To date, this model comparison procedure has been implemented by estimating multiple models and computing the evidence for each, which is prohibitively time-consuming for investigating multiple hypotheses. This paper introduces a more computationally efficient method based on the Savage-Dickey ratio [21], [22]. Before describing the method we review relevant concepts in Bayesian neuroimaging. Readers requiring a more comprehensive background to Bayesian inference are referred to standard texts [23], [24].

### PPMs for Parameter Inference

PPMs are similar to SPMs in that they are also based on a mass univariate approach in which General Linear Models (GLMs) are fitted to data at each voxel [25]. They differ however in the statistical method used to estimate parameters and make inferences. Estimates of the GLM parameters, for example, are constrained using empirical priors.

Early work on Bayesian fMRI considered mass-univariate approaches to modeling spatial dependencies in the signal and noise. For example, Gossl et al. [8] proposed a separable spatio-temporal model where these spatial dependencies were characterized using Markov Random Field (MRF) priors. More recently, Woolrich et al. [18] described a Bayesian model of fMRI in which the noise process was characterized by separable or nonseparable spatio-temporal models. Both of these approaches used Markov Chain Monte Carlo (MCMC) to perform posterior inference, which is computationally expensive.

We have previously proposed a non-spatial PPM procedure employing global shrinkage priors which shrink parameter estimates toward zero [17]. We have additionally developed a PPM approach specifically for within-subject fMRI time series [26]. This allows users to specify either global shrinkage priors, or spatial priors based on Gauss-Markov Random Fields (GMRFs) which constrain effect sizes to be similar at nearby voxels. These models are particularly suited to within-subject fMRI, as the error correlations can be modelled using arbitrary-order voxel-specific autoregressive (AR) models. These AR models accurately describe the physiological noise processes in fMRI data [27]. Later work allows for spatial priors on the AR parameters [20] and the approach has been extended to incorporate spatial priors based on wavelets [28] and Gaussian processes [29].

For the above approaches, the result of the estimation is a posterior distribution of effect size at each voxel, 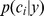, where 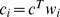 is a linear combination or ‘contrast’ of the GLM parameters at the 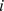
th voxel, 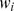. These voxel-wise posterior distributions or PPMs are visualised by specifying two thresholds – an effect size threshold, 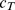, and a posterior probability threshold 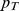 – and plotting voxels for which 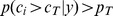. Depending on the software, what is actually plotted can be the posterior probability or the effect size itself. One may also have the option of plotting the log posterior odds, 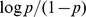, which improves the visualisation for voxels that have posterior probabilities close to unity.

Inferences based on PPMs thus allow researchers to be more specific as to the effects in which they are interested. For example, effect sizes less than 0.1% of the global mean may be deemed physiologically irrelevant (see also a related though less principled method to avoid declaring voxels with trivial effect sizes significant (in a frequentist sense) due to artefactually low variance [30]). An alternative perspective is that needing to specify an additional arbitrary threshold (the effect-size threshold) may be seen as a disadvantage of the method. This has motivated the development of PPMs for model inference.

### PPMs for Model Inference

We first distinguish between nested and non-nested model inference. In nested model inference, a ‘nested’ model can be formulated as a special case of a more general ‘full’ model. For example, nested models may be constructed by removing one or more explanatory variables from the full model. When models are not related in this way they are said to be non-nested. This will be the case if each model has its own unique set or subset of explanatory variables that are not found in the other model.

For non-nested model inference we can proceed by separately fitting the models of interest, computing the model evidence for each, and then plotting a map of the posterior model probability or log Bayes factor. This procedure, which we refer to as Independent Model Optimization (IMO), is straightforward because the evidence of a GLM can be computed exactly [31], [32]. This is not the case for nonlinear models, such as the Dynamic Causal Models used in the study of brain connectivity [1].

This model inference approach has been applied in the context of within-subject models of fMRI time series [20], and allows one to compute a model evidence map; a map of (log) model evidence as a function of space. If one computes a model evidence map for each model of interest, and for each subject in a group, then one can make an inference at the population level as to which model is the most prevalent [33]. The method can accomodate any number of models (not just a null model and a single alternative). This approach has been used, for example, to show that in a forced-choice decision task, anterior brain regions integrate contextual information over longer time periods than do posterior regions [34].

To show that a brain region does not activate requires a strong Bayes factor in favour of the null model over the alternative model for the data in that region. This inference requires the specification of a single parameter, namely what is meant by ‘strong’. Here we can refer to established scales of strengths of evidence [22], [35] where, for example, a Bayes factor of at least 20 (or log Bayes factor of at least 3) corresponds to strong evidence. It is also possible to declare that a region does not activate using PPMs for parameter inference, but this requires specification of an additional parameter - the effect size threshold [17]. The model comparison approach is therefore more parsimonious.

Whilst PPMs based on model inference are a powerful paradigm for the analysis of fMRI time series, they are somewhat computationally demanding, because for every model comparison one wishes to make, it is necessary to fit all models over the spatial domain of interest, and compute the evidence for each. If one has a small region of interest this is less of an issue, but whole-brain analyses can require tens of minutes of fitting time for each model to be considered.

We now describe the special case of nested model comparison. Previously, we have proposed an analogue of the classical F-test, which instead uses a 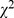 test based on the posterior density [36]. The resulting test is conceptually rather unsatisfactory, however, as it implements a classical inference based on a Bayesian posterior density. This paper proposes replacing the 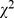 test with an inference based on the Savage-Dickey ratio. As we shall see, this new approach will also provide a computationally efficient method for non-nested model comparison. This extends recent work in brain connectivity analysis where we have proposed [37] and validated [38] a generalisation of the Savage-Dickey approach in the context of Dynamic Causal Modelling [39].

## Methods

This section first describes Bayesian model and parameter inference for the GLM. We then describe the statistical tests for nested and non-nested model comparison including the Savage-Dickey ratio. In our implementation of Posterior Probability Mapping (PPM) we do not store posterior covariance matrices as this would require a prohibitive amount of computer disk space. Instead, we store a small number of hyperparameters to reconstruct the covariance matrices using a Taylor series approximation. This additional step is described in a later subsection. We also show how PPMs can be derived for both first- and second-level models. In what follows 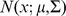 denotes a multivariate Gaussian distribution with mean vector 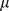 and covariance matrix 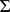, of which 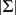 is the determinant.

### Bayesian General Linear Model

We consider Bayesian inference for GLMs with data 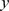, design matrix 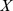 and regression coefficients 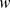. We assume a Gaussian prior over regression coefficients
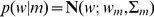
(1)where 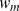 and 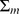 are the prior mean and covariance for model 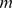. In most applications to fMRI [17], [26] the prior mean is set to zero, and the prior covariance is estimated using multiple time series over a spatial region. This is described in more detail below in the section on Empirical Bayes. The variable 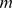 symbolises the model assumptions. Different models are usually thought of as being specified by having different design matrices. In GLMs a single parameter is associated with each column of the design matrix, therefore different models have different parameters. It is also possible to conceive of different models as having different priors, hence the notation above. For example, subspaces of the design matrix can be eliminated by setting the corresponding parameters to have zero prior mean and zero prior variance.

We also assume a Gaussian likelihood
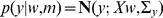
(2)where 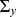 is the observation noise covariance matrix. Like the prior covariance, the noise covariance is typically estimated from the data, as described in the section on Empirical Bayes. Given a Gaussian prior and likelihood, the posterior over regression coefficients is also Gaussian [40]

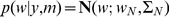
(3)with posterior mean 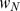 and posterior covariance 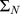 given by



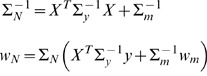
(4)


Bayesian inference over models is implemented by first computing the model evidence 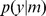. If one has a prior distribution over a set of models, 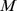, this can be updated into a posterior distribution using Bayes rule and the model evidence
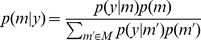
(5)


For pairs of models with equal model priors, 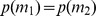, inference can be made based on the Bayes factor [22]. The Bayes factor for model 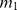 versus 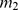 is given by
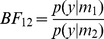
(6)

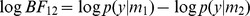



For GLMs, assuming known 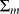 and 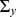, the log model evidence, 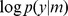, can be computed as
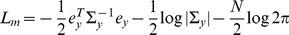
(7)

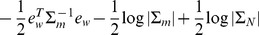
where the ‘prediction errors’ are



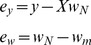
(8)Unequal model priors are accomodated by making inferences using posterior odds ratios, instead of Bayes factors. The posterior odds is equal to the prior odds times the Bayes factor
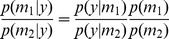
(9)


Taking logs gives
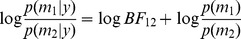
(10)


Thus if 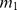 is 100 times less likely a priori than 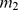, the log posterior odds equals the log Bayes factor minus 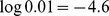. Hence, unequal prior odds can be dealt with by a simple change to the decision threshold.

### Empirical Bayes

We first discuss the approach to second-level fMRI analysis which is described in [17]. This takes an Empirical Bayes approach which estimates parameters of the prior 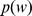 using data from all voxels in the search region. The prior mean is set to zero, 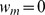, and the prior covariance is assumed diagonal 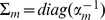 with the 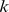
th element of 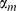 denoting the prior precision of the 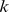
th parameter. The observation noise covariance matrix at the 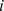
th voxel, is then parameterised as
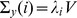
(11)where 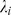 is a single voxel specific hyperparameter and 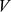 is a matrix which captures the global observation noise structure and has been estimated in a previous step. The hyperparameters 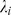 and 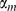 are then set to maximise the model evidence using an Empirical Bayes approach [17]. This optimisation does not require the model evidence itself to be computed.

For first-level models the approach is similar. The main difference is that 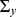 is set to accommodate voxel-wise Auto-Regressive (AR) noise processes of arbitrary order, so as to absorb aliased temporal fluctuations due for example to respiration and heartbeat. Here, 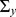 is parameterised using voxel-specific AR parameters. It is possible to set the AR model order to zero, in which case the likelihood reduces to that for the standard GLM. For the first-level models the priors can be either set as ‘global shrinkage priors’, which are identical to the second-level priors described above, or as spatial priors which encourage parameter estimates to be similar at nearby voxels [26]. All the hyperparameters are estimated together, along with the prior precisions using Empirical Bayes [26]. This paper is primarily concerned with evaluation of the Savage-Dickey approach for global shrinkage priors.

For the above Empirical Bayes approaches, the expression for the log model evidence in equation 7 should be augmented with penalty terms to accomodate the uncertainty in the estimation of the associated hyperparameters. These terms are provided, for example, in equations 8 and 13 in [20]. For the results in this paper the inclusion of these extra terms made little or no quantitative difference so, for ease of communication, the IMO results presented in this paper are based on equation 7.

### Nested Model Comparison

This section describes the Savage-Dickey approach for nested model comparison. If model 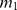 is nested within 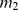 where the models have common parameters 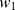 and 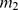 has additional parameters 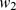, then the Bayes factor can be rewritten as follows. First, we write the evidence for model 2 given that 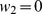


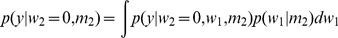
(12)


Because we have a nested model the likelihood term 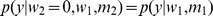. This is the mathematical definition of a nested model. Second, if it is the case that the priors over the common parameters are the same for the two models, 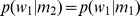, then we can write
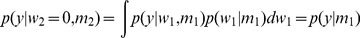
(13)


Substituting into the Bayes factor (equation 6) gives
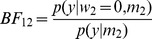
(14)


Using Bayes rule over the posterior of 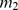 gives
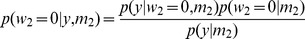
(15)


We can therefore see that
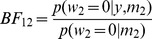
(16)


**Figure 1:**
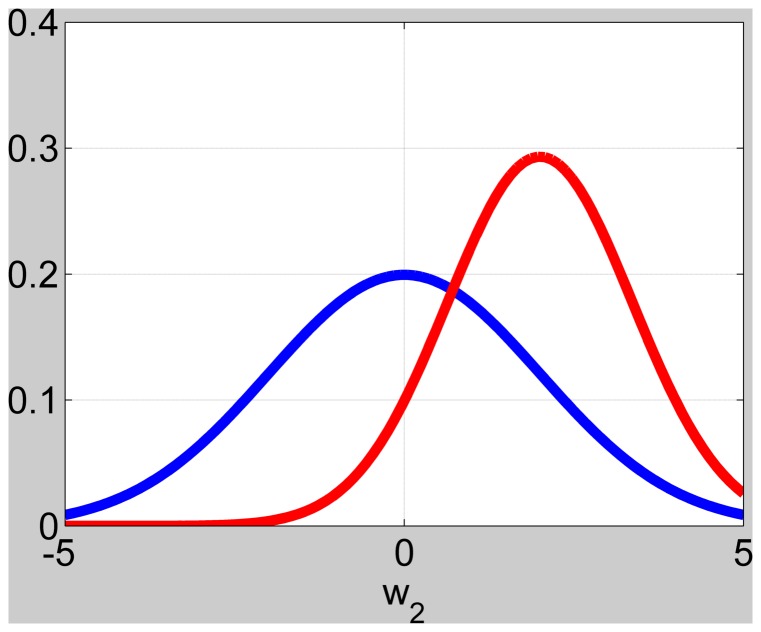
The figure shows the prior density 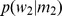 in blue and the posterior density 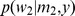 in red. Here 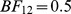, weakly favouring the more complex model 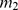, since the parameter 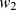 is half as likely to be zero after seeing the data than before.

The formula makes intuitive sense and is known as the Savage-Dickey ratio [21]. If we believe it is more likely that parameters are zero after seeing the data than before, then 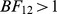 and we have evidence in favour of the nested model. Figure 1 illustrates the opposite case for a simple one-dimensional example. For nested model comparisons the Bayes factor can therefore be computed by fitting just the larger model. If the priors over the common parameters are not the same for two models then a correction factor, based on a sampling approach, can be computed [41].

The above procedure can be generalised to consider non-zero hypothesized values, and nested models defined as subspaces of full models. This is implemented using the usual approach of defining contrasts for linear models [42]. A single contrast vector, for example, can be used to specify a single hypothesis, whereas multiple contrast vectors combined into a matrix can be used to specify a compound hypothesis. For example, if 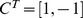 then 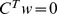 specifies the single hypothesis that 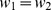. Similarly, if 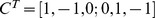 then 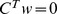 specifies the compound hypothesis that 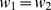 and 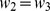. This latter compound hypothesis is rejected if 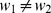 or 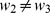. This type of contrast matrix is used, for example, in testing for main effects in factorial designs. More details on hypothesis testing in linear models can be found in standard textbooks [42].

We now consider the use of contrasts for the case of Gaussian priors and posteriors. The Savage-Dickey approximation to the Bayes factor in favour of the alternative hypothesis (full model) over a particular null hypothesis (nested model 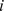) is given by
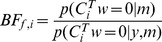
(17)where 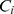 is a contrast matrix and 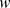 are the regression coefficients. The Savage-Dickey ratio compares the probability density for the null hypothesis under the prior versus under the posterior. If it is a-posteriori less likely then 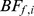 will be large, favouring the full model (as shown in Figure 1).

Given that the prior and posterior are both Gaussians this can be evaluated as
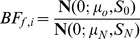
(18)where



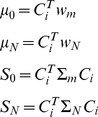
(19)If the prior mean is 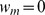 (as it is for Bayesian GLMs implemented in SPM [43]) the log Bayes factor simplifies to
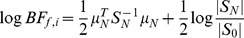
(20)


The Savage-Dickey ratio is exact if 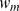, 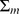 and 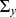 are identical for the two models being computed. Under these conditions it will give identical results to IMO. Most practical implementations of Bayesian inference for fMRI, however, set 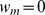 and use an Empirical Bayes procedure to estimate 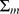 and 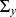. These parameters will therefore differ between models.

Consider, for example, the estimation of 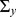 when comparing a simple and complex model. If the simpler model is true then the error variances are likely to be very similar, whereas if the complex model is true then the error variances for the complex model are likely to be smaller. A redeeming feature of error variance estimation, however, is that these estimates are corrected for the degrees of freedom in the model. The effect of Empirical Bayes estimates is addressed empirically at the beginning of the results section.

### Non-nested Model Comparison

The previous section has shown how to compute 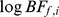 which is the log Bayes factor of the full model with respect to the reduced model defined by contrast 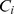. We can also consider a second contrast 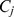 and its associated term 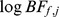. Note that the contrasts 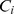 and 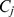 can define two separate subspaces of the full model, for example, by loading onto different sets of regressors in the design matrix. This means that model 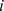 need not be nested within model 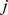 or vice-versa. The only requirement is that both are nested within the full model 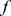.

One can then combine the two log Bayes factors to get 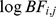 thus providing a procedure for the comparison of non-nested models. We have
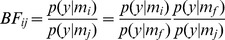
(21)


Hence
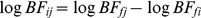
(22)


This idea has been proposed in the Bayesian model selection literature [22] and has been employed [37] and validated [38] in the context of Dynamic Causal Models.

### Group Analysis

The implementation of non-nested model comparisons is based on the log Bayes factor images created as previously described. One can then compute differences in these, as indicated in equation 22, and enter these difference images into a group analysis. For nested model comparisons the log Bayes factor images, computed using equation 20, can also enter a group analysis in the same way.

To make model inferences regarding the population from which subjects were drawn one can use the same random-effects (RFX) model selection procedure as described previously [33]. Here the ‘random-effect’ is a discrete variable which indexes which model each subject uses. This presents an alternative to the standard group analysis which implements a random effects analysis over the parameters of a model. This RFX parameter inference procedure is described in standard references [25] and makes use of ‘second-level’ models.

RFX parameter inference looks for group effect sizes which are consistent in relation to the between-subject variability whereas RFX model comparison looks for the models which have the highest frequency in the population. If some subjects show strong negative and others strong positive effects then this could be detected with RFX model comparison but not with RFX parameter inference. Conversely, if there is a consistently signed but small effect RFX parameter inference may be more sensitive.

### Taylor Series Approximation

In our implementation of the above Bayesian estimation algorithms, the full voxel-wise posterior covariance matrices are not explicitly stored as this would require a prohibitive amount of disk space. For GLMs with 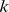 parameters each covariance matrix comprises 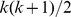 real numbers. For brain images comprising 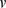 voxels this gives a total of 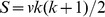 real numbers to store. For example, for 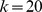 and 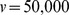 we have 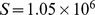 or 210 images. Instead we store a small number of parameters that allow us to reconstruct these covariance matrices using a first-order Taylor series approximation. For example, in the ‘second-level’ PPM approach [17] the posterior covariance (4) at voxel 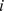 depends on 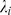 via the noise covariance (11),
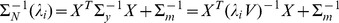
(23)where 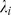 is a single voxel specific hyperparameter and 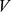 is a matrix which captures the observation noise structure and has been estimated in a previous step [17]. These hyperparameters 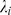 are the same quantities referred to in the above section on Empirical Bayes. Viewed as a function of a continuous parameter 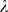, 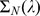 can be analytically differentiated, allowing the posterior covariance to be approximated using a first-order Taylor series
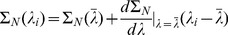
(24)where 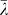 is the mean hyperparameter averaged over the volume of interest. Thus we need only store a single voxel specific hyperparameter, 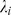, 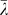 and the single Jacobian matrix 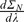 evaluated at 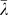. Thus for 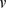 voxels the total storage required is 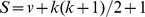. This breaks down as 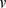 for the 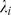, 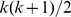 for the Jacobian and one for 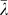. For our numerical example this gives 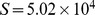 or between 1 and 2 images. This requires less storage by a factor of over 200.

A similar Taylor series approach is used for first-level models [44]. The fact that we will not be using the exact posterior distributions to compute the Savage-Dickey ratios in equation 20 will create an extra level of approximation in the computation of log Bayes factors. We therefore refer to the overall approach as the Savage-Dickey-Taylor (SDT) method.

### Summary

We have described the use of Savage-Dickey ratios initially for the case of nested model comparisons. This brings about a natural symmetry with classical inference based on SPMs. For SPMs there are two types of test. The SPM-t allows one to test for one-sided effects. The Bayesian analogue of the SPM-t is the PPM for parameter inference. The SPM-F allows one to test for two-sided effects for both uni-dimensional or multi-dimensional contrasts (the contrast matrix 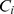 has a single row, or multiple rows). The Bayesian analagoue of this is the Savage-Dickey test for a nested model comparison.

We have also shown how the Savage-Dickey approach can be used for non-nested model comparison. Importantly, whether the comparison is nested or non-nested the computational saving is great, because we only need to estimate a single full model. To save storage space, practical implementations of these Bayesian algorithms reconstruct posterior parameter covariance matrices using a Taylor series approach. We therefore describe our overall approach as the Savage-Dickey-Taylor (SDT) method. In what follows we compare the proposed SDT method for model inference with the previously proposed Independent Model Optimization (IMO) approach, which requires separate fitting of full and nested models.

### fMRI Data

We present first- and second-level analyses of data from an fMRI study of face processing. The data were collected to study neuronal responses to images of faces and are available from the SPM web site [43]. For a full description of this data set and similar analyses see [45]. Each face was presented twice and faces belonged to either familiar (‘F’) or unfamiliar (‘N’) people which gave rise to four conditions (‘N1’, ‘N2’, ‘F1’, ‘F2’). For the first-level analyses hemodynamic responses were modelled with a single ‘canonical’ hemodynamic basis function [43]. Together with a constant column, this gives rise to a design matrix containing five columns which we refer to below as the ‘standard’ first-level model. We use this standard model to analyse data from a single subject.

The second-level analysis (RFX parameter inference) proceeds as follows. Data from 12 subjects were first analysed using 12 separate first-level models. These were not the standard model, as above, but treated all face presentations as a single event type. Responses were then modelled using a 12 time bin Finite Impulse Response (FIR) model as described in the Group analysis section of the SPM manual. Each time bin was 2 s wide thus covering a 24 s post-stimulus epoch. First-level contrasts were then used to produce summary statistic images for each time bin and for each subject. This resulted in 144 images which were used as data for the second-level models described in the results section below.

## Results

We first investigated the accuracy of the Savage-Dickey (SD) approximation using simulation studies to assess the effect of empirical estimation of observation noise and prior precision. We also assess the effect of the Taylor approximation. We then report the accuracy of SDT versus IMO on empirical first- and second-level fMRI data.

### Observation Noise

As noted in the theory section, SD is exact if the likelihoods, and therefore the obervation noise parameters, are the same between models. However, in practice the observation noise parameters are estimated from the data. Our simulations examined the effect of this estimation on the accuracy of the approximation.

We defined a ‘reduced model’ corresponding to the standard first-level model design described above. This has four regressors of interest, one for each of the four experimental conditions and an uninteresting constant column. We then defined a ‘full model’ which had these regressors, but in addition had two columns for parametric modulators. These modulators modelled responses as exponential functions of the lag between first and second presentations of face image 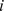, in terms of the number of intervening faces. The exponential function was given by 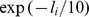 where 10 denotes the chosen time constant (in units of number of faces presented).

**Figure 2:**
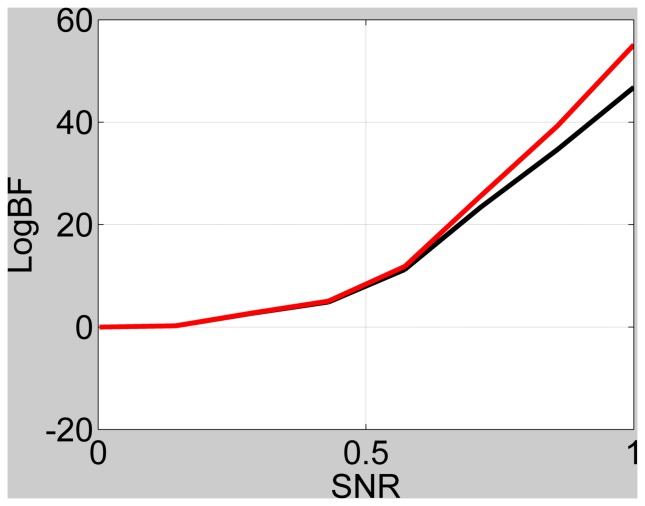
Log Bayes factor versus SNR for full versus reduced model, when full model is true, for IMO approach (black line) and Savage-Dickey (red line).

**Figure 3:**
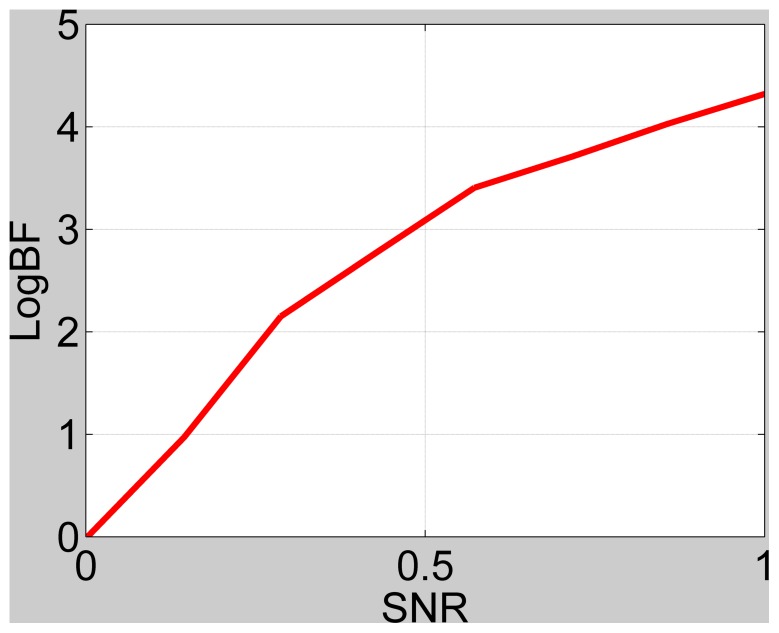
Log Bayes factor versus SNR for full versus reduced model, when reduced model is true, for IMO approach (black line) and Savage-Dickey (red line). The lines overlap.

We generated data sets with a range of signal-to-noise ratios (SNRs) similar to the simulations in [32]. Here SNR is defined as the ratio of signal standard deviation to noise standard deviation. Figure 2 shows the simulation results for the case of data generated from the full model, and Figure 3 for data generated from the reduced model. For the latter case, SD is almost exact as the noise estimates converge to the same values for full and reduced models. For the former case, SD becomes biased at high SNR because the observed noise is over-estimated for the reduced model due to the presence of unmodelled signals. However, this only occurs at very large values of log Bayes factor (favouring the full model) so is unlikely to have any practical effects on the resulting inference.

### Prior Precisions

This simulation generates data from a design matrix that is similar to many second-level models. We use a design matrix 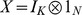 which models K effects using data from 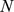 subjects. This corresponds to a One-way ANOVA design with K levels. For the simulations we set 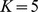 and 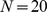. We specify a prior over regression coefficients to have zero mean and precision 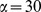 for each coefficient. The observation noise precision was set to 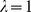. We first draw the regression coefficients, 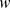 from this prior and produce data using 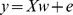 where 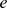 has zero mean and precision 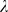. We draw data at 1000 simulated voxels.

We then test for the effect of the first two regressors using the contrast
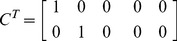
(25)


The null model corresponding to this has design matrix 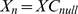 where 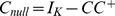 [42]. For the above contrast we have
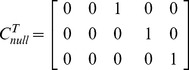
(26)


The SD log Bayes factor is computed using equation 20 using the true observation noise 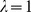. Instead of using the true 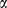
’s we use a modified set of alphas. We draw 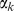 (for the 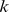
th regression coefficient) from a uniform distribution between plus and minus 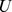
% percent of 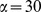. This mimics the variability introduced by the Empirical Bayes estimates of the 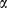
’s.

We then compute the IMO log Bayes factor by separately computing the model evidence for the full and null models. Again, we use the true observation noise 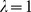 but use a modified set of alphas. Here, the alphas for the full model are the same as for the SD simulation above. But the alphas for the null model are adjusted using the same uniform sampling approach to produce a different set of 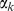
’s. This reflects the fact that the Empirical Bayes IMO approach uses two different sets of alphas; one for the full model and one for the null model.

**Figure 4:**
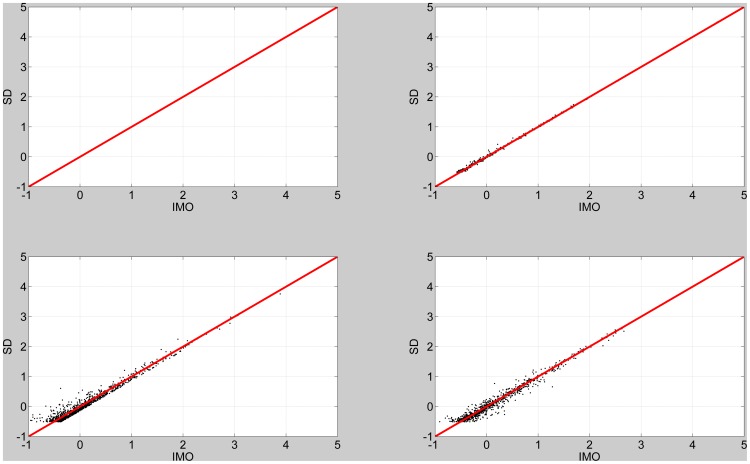
Second-level design. Savage-Dickey log Bayes factor versus IMO log Bayes factor for four levels of variability in prior precisions: 0% (top left), 17% (top right), 33% (bottom left) and 50% (bottom right). The red line denotes equality.

We repeat the above procedure for four levels of variability in the prior precisions; 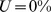, 17%, 33% or 50%. Figure 4 shows SD versus IMO estimates of the log Bayes factor for these four different levels. For all modifications of prior precisions, larger log Bayes factors are accurately approximated. There is, however, increasing levels of disagreement at the lower range. The most noticeable feature is a ‘bottoming-out’, most clearly observable for the 50% condition. This occurs because the IMO estimate is a function of two sets of 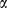
’s (full and null model) whereas the SD estimate is only a function of one set of 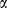
’s (full model).

For null prior precisions which are smaller than full prior precisions, the IMO estimate is more negative - hence the dots left of the red line in Figure 4. Null prior precisions larger than full prior precisions produce dots to the right of the red line. Similar results have been obtained (not shown) when using contrasts testing for additive or differential effects.

**Figure 5:**
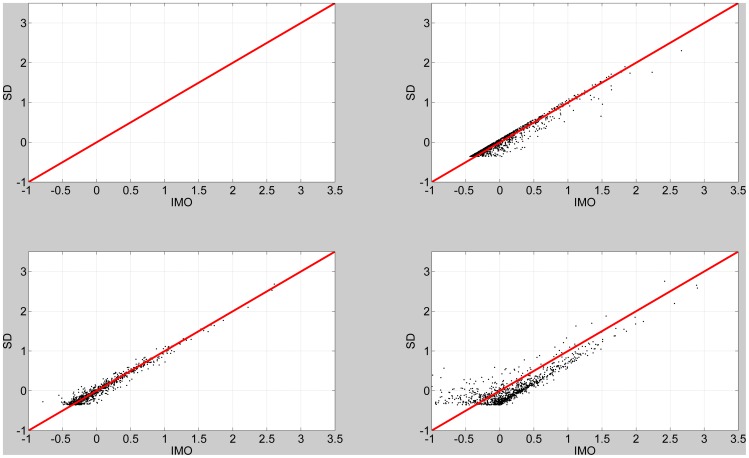
First-level design. Savage-Dickey log Bayes factor versus IMO log Bayes factor for four levels of variability in prior precisions: 0% (top left), 17% (top right), 33% (bottom left) and 50% (bottom right). The red line denotes equality.

We repeated the above procedure but this time using the standard first-level fMRI design matrix. An observation noise precision of 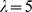, which is representative of values estimated from event-related fMRI data (see below), was set to be the same for both models. The results are shown in Figure 5 for a contrast testing for a differential effect. Again, we observe a bottoming-out effect. Further simulations showed that the bottoming-out effect could be produced for first- or second-level designs, and for subset, differential or additive contrasts. This effect could be alleviated by setting the observation noise precision to a sufficiently high level. To summarise, SD and IMO agree well for moderately positive IMO log BFs. But for negative IMO log Bayes factors, the discrepancy becomes commensurately larger for decreasing observation noise precision and increasing heterogeneity of the prior precision estimates.

**Figure 6:**
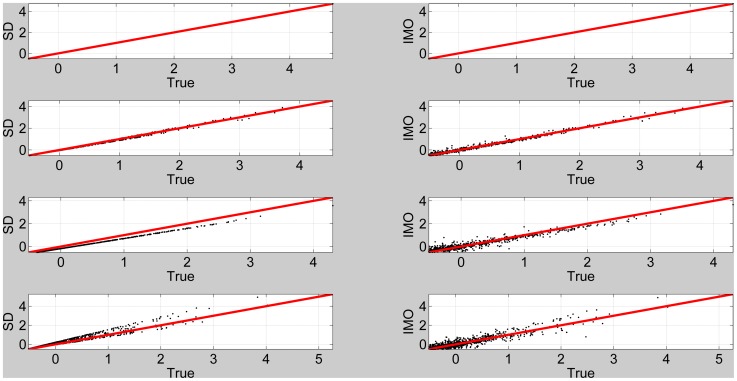
Left Panel: Savage-Dickey log Bayes factor versus true log Bayes factors for four levels of variability in prior precisions. Right Panel: IMO log Bayes factor versus true log Bayes factors for four levels of variability in prior precisions. These are 0% (first row), 17% (second row), 33% (third row) and 50% (last row).

Finally, we compare SD and IMO estimates to the true log Bayes factors. In these simulations, regression coefficients were sampled from distributions with known prior precision (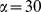, as above) and this value was used to compute the true log Bayes factor. IMO estimates were based on full prior precisions and null prior precisions that were both modified by a maximum proportion 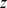. The SD estimates were based on the modified full prior precisions. Bayes factors were then computed for 1000 data sets and produced the results in Figure 6. Here we can see that SD and IMO produce different patterns of errors in their estimation, with SD showing a degree of bias and IMO showing a degree of variance. We then computed the Root Mean Squared Error (RMSE) in estimating the log Bayes factor for the above results. This procedure was repeated 100 times. For 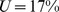, 33% and 50% the RMSE’s are 0.07, 0.14 and 0.24 for SD and 0.07, 0.15 and 0.25 for IMO. There is therefore very little difference in the average accuracy of the estimates.

### Taylor Approximation

We repeated the ‘first-level’ fMRI simulations described in the above section on observation noise. But this time we hold the noise precision fixed and look at the effect of approximating the posterior density using the Taylor series approximation. We used empirical values of observation noise levels from 2000 voxels of first level fMRI data taken from slice 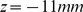 (see below). These ranged from 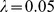 to 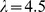. We compared the log Bayes factors as estimated using SDT versus SD over 2000 simulated voxels and found excellent agreement. The SDT estimates were within 0.00007%, 0.00009% and 0.00022% of the SD values, for AR model orders of 1, 2 and 3 respectively. Plots of SDT and SD versus SNR (not shown) are visually indistinguishable.

### First-level fMRI

We first fitted the first-level models using the ‘1st level’ Bayesian estimation algorithm described in [20] using a ‘global’ prior. We additionally constrained the analysis to within brain voxels using an explicit mask (the brainmask.nii image in SPM’s apriori directory). Model fitting took 6 minutes on a high-end desktop PC (dual 3.2 GHz Intel Xeon CPUs, 12 GB Ram, 64-bit Windows Vista).

We used the SDT approach to compute Bayes factors testing for responses to non-familiar images (the fifth column of zeroes in the contrast relates to the uninteresting constant column in the design matrix, and is often not explicitly included when defining contrasts in the SPM software)
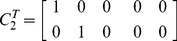
(27)


The log Bayes factor at each voxel was computed using equation 20. We also computed 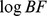 maps using the IMO approach by fitting two models. First, we fitted the standard model and computed its log evidence, 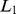, at each voxel using equation 7. Second, we fitted a reduced model which did not model responses to non-familiar faces. Thus, the reduced model has three regressors whereas the standard model has five. We then computed the log evidence map 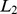. The log BF map testing for responses to non-familiar faces is 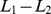. The models were estimated using the ‘1st level’ Bayesian estimation algorithm described in [20] using a ‘global’ prior. Model fitting took 14 minutes for the standard model and 12 minutes for the reduced model. Each estimation took longer for the IMO approach because the model evidence was computed at each voxel.

**Figure 7:**
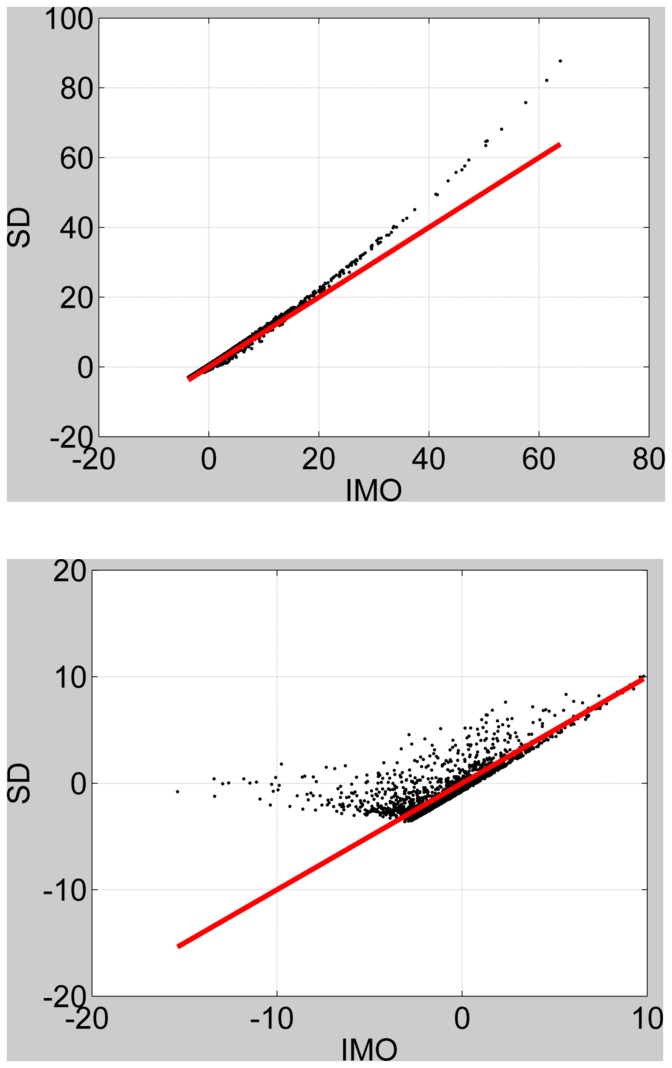
First-level models. Top Panel: log Bayes factor for SDT versus IMO approaches testing for any response to non-familiar faces. The red line denotes equality. Bottom Panel: log Bayes factor for SDT versus IMO approaches testing for main effect of familiarity



Figure 7 (top panel) plots SDT versus IMO log Bayes factors for voxels in slice 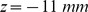. This shows good agreement, except at large values of IMO log Bayes factor. The overall correlation is 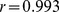. The plot shows a similar effect to that observed in Figure 2, suggesting that the discrepancy may be due to inconsistent estimates of observation noise precision. We then repeated the above analysis but with the contrast now testing for the main effect of familiarity.

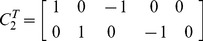
(28)This tests for differences between responses to familiar versus unfamiliar faces, collapsed across repetition. Figure 7 (bottom panel) plots SDT versus IMO log Bayes factors for voxels in slice 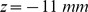. This shows poor agreement over a large range of IMO log Bayes factor values. The overall correlation is 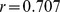. The plot shows a similar effect to that observed in Figure 5, suggesting that the discrepancy may be due to inconsistent estimates of prior precision in the context of large observation noise.

### Second-level fMRI

**Figure 8:**
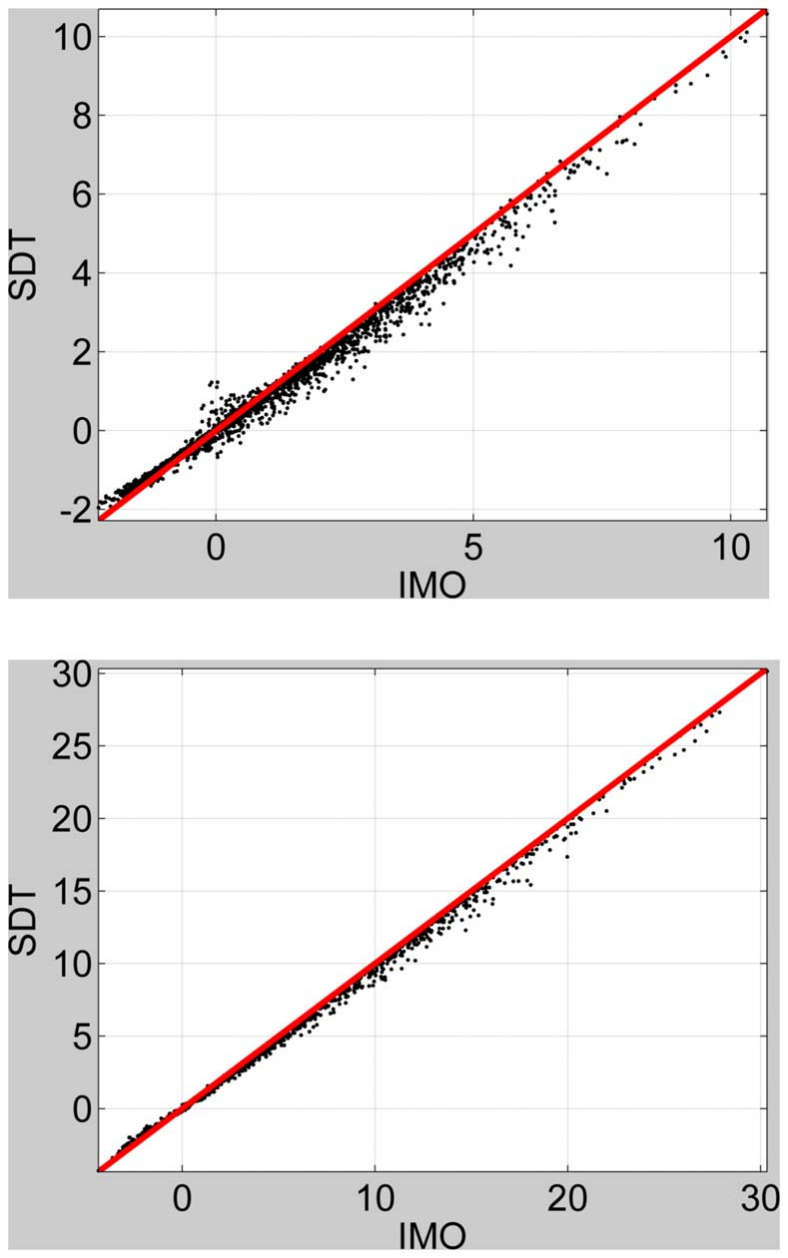
Second-level models. Top Panel: log Bayes factor for SDT versus IMO approaches testing for response in 6 to 12 s time bins. The red line denotes equality. Bottom Panel: log Bayes factor for SDT versus IMO approaches testing for responses that are better explained by a 4 to 6 s model than a 6 to 8 s model.

We fitted a second-level model to the FIR summary statistic images as described earlier using the global shrinkage prior approach [17]. This was a one-way ANOVA model with a single time-bin factor. We then used SDT to compute the log Bayes factors for comparing the standard model to a nested model which did not include responses in the 3 time bins from 6–12 s. This was implemented using equation 20 and the appropriate contrast (an identity matrix over columns 4, 5 and 6). We then estimated this log Bayes factor using the IMO approach by separately fitting the standard and reduced models and computing the model evidences using equation 7. Figure 8 (top panel) plots the log Bayes factors for SDT versus IMO approaches for voxels in the 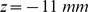 slice. This shows a very strong correlation between the measures (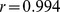). Our decision to look for late responses, in the 6–12 s window, is rather arbitrary but we note that similarly good agreements between SDT and IMO were found for other time windows.

We also implemented a non-nested model comparison to find where in the brain BOLD responses were better explained by a 4 to 6 s bin model versus a 6 to 8 s bin model. This model comparison looks at the relative amounts of variance explained by the different models, and is not the same as a contrast testing for a difference in the mean response in each bin. This test was first implemented using the SDT approach by specifying the two contrasts and subtracting the resulting log Bayes factor images using equation 22. This was then compared to the IMO approach where we separately computed the evidence for each model. We then plotted the log Bayes factors for SDT versus IMO approaches in Figure 8 (bottom panel). This figure is for voxels in the 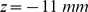 slice. This shows a very strong correlation between the measures (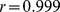). Similarly good agreements were found over a range of time bin comparisons.

## Discussion

Statistical Parametric Mapping (SPM) has become the dominant paradigm for mass-univariate analysis of neuroimaging data. This paper has examined an alternative Posterior Probability Mapping (PPM) approach which offers two advantages (i) inferences can be made about effect size, thus lending a precise physiological meaning to activated regions, (ii) regions can be declared inactive. This latter facility is most parsimoniously provided by PPMs based on Bayesian model comparisons. Previously, these comparisons have been implemented by an Independent Model Optimization (IMO) procedure which separately fits null and alternative models. In this paper we have proposed a more computationally efficient method based on Savage-Dickey approximations to the Bayes factor and Taylor series approximations to the voxel-wise posterior covariance matrices.

The IMO approach is more time consuming both due to the time taken to estimate the models and the user’s time taken to set up the relevant design matrices. The Savage-Dickey-Taylor (SDT) approach is quicker on both counts and allows the user to explore the model space in a truly interactive way which is analagous to the use of F-contrasts in classical inference. Simulations show that the accuracy of the SDT method is comparable to that of the IMO method. Results on fMRI data show a correlation between SDT and IMO estimates, that is consistently high for second-level data, but is only moderately high for first-level data.

Our current Empirical Bayes implementation for estimating prior precisions works slice-by-slice for first-level data, due to computational constraints, but over the whole volume for second-level data. This has the effect of rendering the estimates of prior precisions more variable for the first than the second-level. The results in this paper suggest we revisit this implementation. Until these first-level estimates have been re-implemented we recommend that SDT only be used at the second level.

In general, the SDT approach would be suitable for all neuroimaging modalities. However, in this paper we have only implemented it for the case of global shrinkage priors; these are appropriate for fMRI because the null hypothesis is of no activity on average [17]. For PET and M/EEG, when processed so that the data features represent activation (or, more generally, differences between conditions, whose expectation is zero under the null hypothesis) the methods presented here are similarly appropriate.

However, some modalities have imaging data that would not be zero under the null, such as voxel-based morphometry (VBM), whose voxel-wise data represent local tissue volumes [46] or forms of PET with a single image per subject that does not represent a difference between conditions, for example amyloid imaging [47]. For these data, shrinkage of the voxel-wise parameter estimates towards a *non-zero* overall mean should be appropriate and straightforward. We will therefore examine the use of SDT for these non-zero mean priors in a future publication. This future work will also extend SDT to work with spatial priors [20]. Both of these extensions are mathematically straightforward but beyond the scope of the current paper.

### A Software Implementation

Many of the algorithms referred to in this paper are available in the SPM software package which is available from http://www.fil.ion.ucl.ac.uk/spm/. The PPM procedure employing global shrinkage priors which shrink estimated parameters towards zero [17] can be accessed in the user interface of SPM by choosing ‘2nd-level’ fMRI or M/EEG models and selecting the Bayesian option. The PPM approach for the analysis of within-subject fMRI time series [26] can be accessed in the user interface of SPM by choosing ‘1st level’ fMRI models and selecting the Bayesian option.
